# Effect of Pre-Oxidation Treatment on Corrosion Resistance of FeCoSiBPC Amorphous Alloy

**DOI:** 10.3390/ma15093206

**Published:** 2022-04-29

**Authors:** Qian Zhang, Xidong Hui, Zongzhen Li, Guangqiang Zhang, Jingcheng Lin, Xuelian Li, Wei Zheng, Xia Cao, Shaoxiong Zhou

**Affiliations:** 1 State Key Laboratory for Advanced Metals and Materials, University of Science and Technology Beijing, Beijing 100083, China; atzhangqian@163.com (Q.Z.); hy.zw@163.com (W.Z.); 2 Central Iron and Steel Research Institute, Beijing 100081, China; 3 Jiangsu JITRI Advanced Energy Materials Research Institute Co., Ltd., Changzhou 213001, China; zongzhenli@hotmail.com (Z.L.); 18601017215@126.com (G.Z.); 4 Department of Electrical & Information Engineering, Shandong University, Weihai 264209, China; jclin1997@163.com (J.L.); hunterlxl@163.com (X.L.); 5 Department of Mechanics and Surface Engineering, Changzhou Institute of Technology, Changzhou 213032, China; caox@czust.edu.cn

**Keywords:** amorphous alloy, pre-oxidation, corrosion resistance, oxidation layer

## Abstract

In this paper, the corrosion resistance of FeCoSiBPC amorphous alloy after pre-oxidation and non-oxidation heat treatment is investigated. The corrosion behaviors of Fe_80_Co_3_Si_3_B_10_P_1_C_3_ amorphous alloys in 1 mol/L NaCl solution were investigated by the electrochemical workstation. The pre-oxidation heat treatment can improve the corrosion resistance of FeCoSiBPC amorphous alloy through an increase in the Ecorr value from −0.736 to −0.668 V, which makes it easy to reach a passive state. The corroded morphology and products of amorphous alloys were tested by scanning electron microscopy (SEM) and transmission electron microscopy (TEM). The SEM/TEM analysis showed that, after pre-oxidation treatment, the oxide layer was divided into two layers: the inner layer was amorphous, the outer layer appeared crystalline, and the main oxide was Fe_2_O_3_. During the oxidation process, Co and P elements diffused from the inner layer to the outer layer, forming phosphorus and cobalt oxides with high corrosion resistance on the surface of the ribbon, thereby improving the corrosion resistance of the ribbon.

## 1. Introduction

Amorphous alloy is a kind of metal alloy with a long-range disordered and short-range ordered structure, which has a high saturation magnetization, high permeability, low coercivity, low loss, and other excellent magnetic properties. At the same time, it also demonstrated high strength, good elasticity, corrosion resistance, and other good mechanical properties, due to it having no dislocation and grain boundary defects. It is widely used in aerospace, the power sector, electronics, automobiles, new energy, and other fields [1,2].

Among amorphous alloys, Fe-based amorphous alloys have been widely used in various fields as new energy-saving, environment-protecting, and green materials due to their remarkable soft magnetic properties and low preparation cost. At present, the main amorphous alloys on the market are FeSiB and FeSiBC, which have good amorphous-forming abilities and high saturation magnetic induction intensities [3]. The industrial production of amorphous alloy ribbons is basically carried out in air, and most of their heat treatment is carried out in a low vacuum or even air. Therefore, the oxidation performance is key to their application. In production and application, the addition of a variety of elements will lead to an increase in chemical activity on the surface of the amorphous alloy, resulting in an oxidation corrosion phenomenon in varying degrees, which will change the properties of the amorphous alloys [4]. Equipment that contains amorphous materials may work in environments mixed with high temperature and humidity [5] such as motors, transformers, etc. The oxidation corrosion of amorphous materials will affect the performance, life, and reliability of the equipment. FeCoSiBPC, as a recently developed high Bs amorphous alloy, has a good market application prospect. However, the addition of various elements increases the chemical activity of the surface of the amorphous alloy, which is more likely to cause surface oxidation, thereby changing the properties of the amorphous alloy.

Therefore, it is urgent to explore the oxidation and corrosion behavior of FeCoSiBPC amorphous alloys in different heat treatment environments and establish the relationship between oxidation and corrosion. In this paper, the effect of oxidation behavior on the corrosion resistance of strips was investigated, and the relationship between surface oxidation and corrosion resistance was established. This research has certain guiding significance for the FeCoSiBPC amorphous alloy in the non-vacuum heat treatment process.

## 2. Materials and Methods

**Figure 1:**
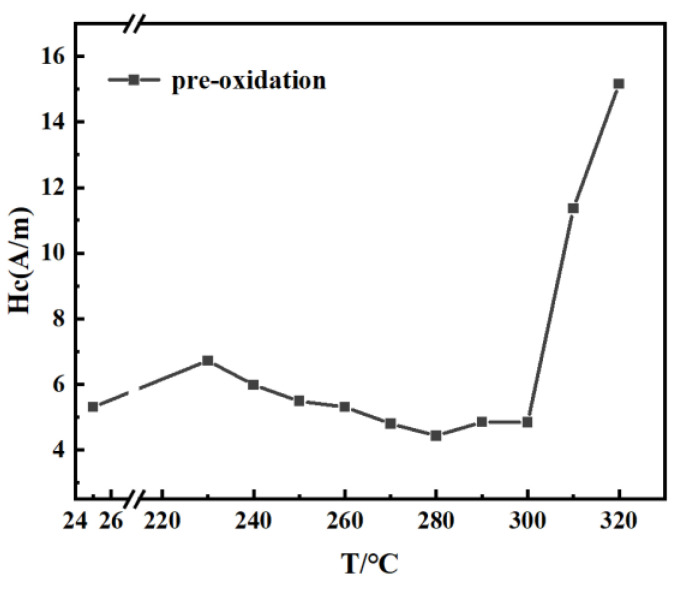
Hc value of 10 min pre-oxidation treated samples.

Multi-component alloy ingots with nominal compositions of Fe_80_Co_3_Si_3_B_10_P_1_C_3_ were prepared by induction-melting the mixtures of high pure Fe (99.99% wt.%), Co (99.99% wt.%), Si (99.99% wt.%), pre-alloyed Fe–C (5.0 wt.% C) ingots, pre-alloyed Fe–P (16.5 wt.% P), and a pre-alloyed Fe–B ingot (17.6 wt.% B) in a purified argon atmosphere. Amorphous alloy ribbon was fabricated by a single copper roller melt-spinning method in an argon atmosphere. The width of the amorphous ribbon was about 5 mm. The thickness of the amorphous ribbons was approximately 20 μm. In order to determine the heat treatment temperature for the best soft magnetic properties of the ribbon, the ribbon was heat-treated for 10 min at 230–320 °C in vacuum and non-vacuum conditions. The non-vacuum heat treatment process was carried out in the atmosphere. The annealing treatment of the amorphous ribbon is to remove the ribbon stress. The optimal heat treatment temperature should be lower than *T_x_*_1_ to avoid the ribbon becoming brittle due to crystallization during the annealing process. The coercivity measurement is shown in Figure 1. The results show that the optimum temperature for the amorphous ribbon was 280 °C. Pre-oxidized and unoxidized samples were obtained by heat-treating the amorphous ribbons at 280 °C for 10–90 min under vacuum and non-vacuum conditions.

Electrochemical experiments were performed on the free side of the tape, and the other side was sealed with ethyl acetate. To verify the effect of the oxide layer on the corrosion resistance of the ribbon, dilute hydrochloric acid (HCL) was used to remove the yellow oxide layer on the surface of the ribbon. The corrosion products of hydrochloric acid were removed with alcohol, then washed with ultrapure water, and dried quickly with cold air for electrochemical experiments.

A VMP3 electrochemical workstation was used to determine the corrosion performance of the amorphous samples after pre-oxidation and vacuum heat treatment. The corrosion solution was 1 mol/L NaCl solution. A Calomel electrode was used as the reference electrode, a platinum electrode was used as the auxiliary electrode, and the sample was used as the working electrode. The result was analyzed by the EIS analysis software Zsimpwin (AMETEK, Berwyn, PA, USA). The MSXD-3 X-ray diffractometer, which was produced by Beijing Beida Smart Microstructure Analysis and Testing Center Co., Ltd. (Beijing, China), was used for XRD analysis. The scanning angle was 10°–90°, and the Cu Kα target was used. The wavelength was λ = 1.54056 nm. The magnetic properties were measured using a Japanese EXPH-100 B-H measuring instrument. Thermal analysis was performed by Mettler Toledo TGA/DSC (Columbus, OH, USA) at a heating rate of 40 K/min. Nova NanoSEM 450 (Nebraska Center for Materials and Nanoscience, Lincoln, NE, USA) scanning electron microscopy (SEM) was used to observe the surface morphology. FEI Strata 400S (FEI, Lausanne, France) focused ion beam scanning electron microscopy (FIB-SEM) was used to cut the ribbon, and FEI Talos F200s (FEI, Lausanne, France) transmission electron microscopy (TEM) was used to observe the cross section of the ribbon.

## 3. Results and Discussion

**Figure 2:**
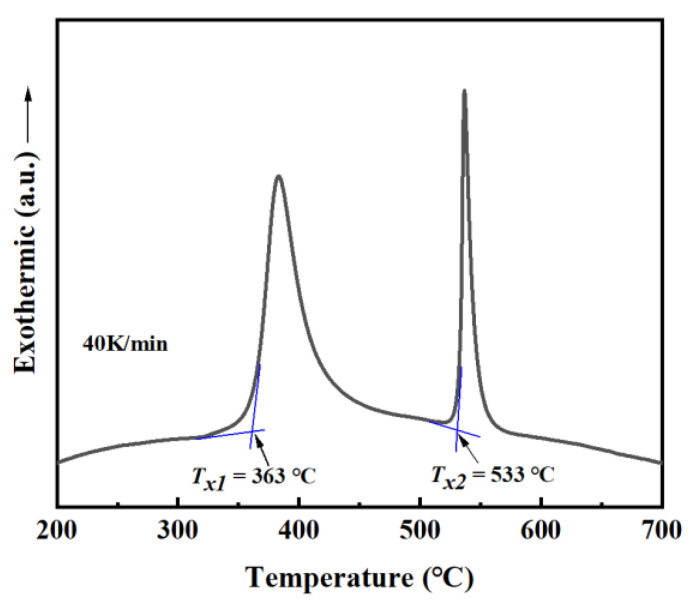
The DSC and TG curve of FeCoSiBPC amorphous alloy.

The crystallization process of the melt-spun Fe(Co)SiBPC ribbon was investigated by DSC at a heating rate of 40 °C/min. As shown in Figure 2, two obvious exothermic peaks corresponding to two different crystallization phases were detected. According to our previous research, the first exothermic peak ($T_{x1}$) corresponded to the crystallization of a–Fe phase, and the second exothermic peak ($T_{x2}$) corresponded to that of Fe–P, Fe–B hard magnetic compounds.

**Figure 3:**
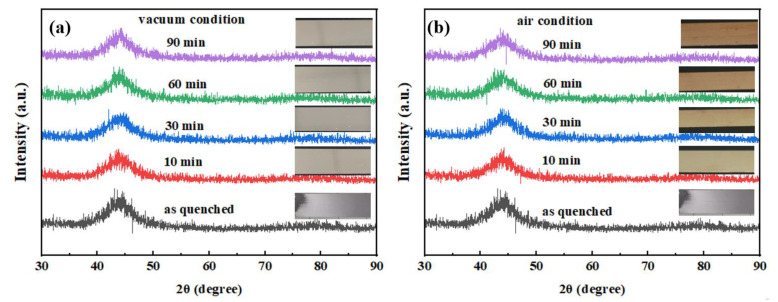
XRD curve and pictures of the FeCoSiBPC amorphous alloy at 280 °C; (**a**) vacuum; (**b**) non-vacuum.

Figure 3 shows the XRD patterns of an FeCoSiBPC amorphous alloy after heat treatment in vacuum and non-vacuum for 10–90 min at 280 °C. It can be seen that the XRD curves of the samples after vacuum and non-vacuum heat treatment showed a good steamed-bread peak shape, indicating that the thin ribbons after heat treatment were still amorphous. Judging from the surface color of the heat-treated ribbon, the vacuum heat-treated samples maintained good metallic luster without oxidation. The color of the non-vacuum heat-treated ribbon gradually deepened with the prolongation of heat treatment time, indicating that the degree of oxidation became gradually severe. Because the X-ray penetration depth of XRD phase analysis was limited, it indicated that the thickness of the oxide layer on the surface of the ribbon was extremely thin.

**Figure 4:**
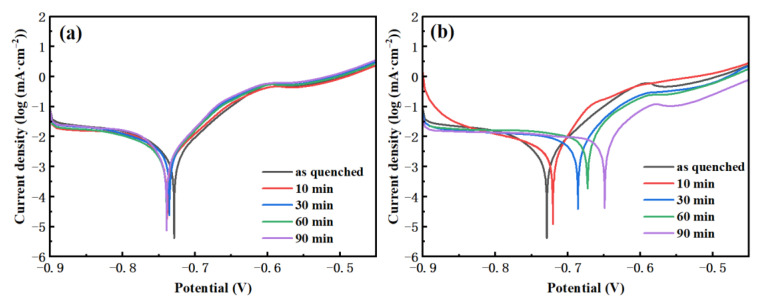
The polarization curves of FeCoSiBPC amorphous alloy at 280 °C; (**a**) vacuum; (**b**) non-vacuum.

Figure 4 shows the polarization curves of the FeCoSiBPC amorphous alloy treated by vacuum and non-vacuum in a 1 mol/L NaCl solution at room temperature. It is seen in Figure 4a,b that all samples had a tendency for passivation. A passivation platform appeared at around −0.575 V, indicating that a passive film was formed. The minimum value (peak value) in the figure represents the corrosion resistance potential of the amorphous ribbon. The polarization curves of the samples after vacuum heat treatment are very close to the as-quenched sample, and the corrosion potential peaks moved slightly to the left. The polarization curves of pre-oxidation treated samples are obviously different. The corrosion potential peaks moved to the right, which means an improvement in the corrosion resistance.

**Figure 5:**
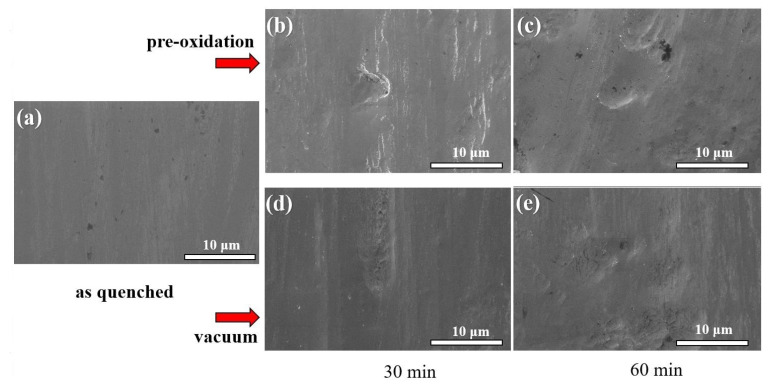
The surface morphologies after heat treatment at 280 °C for different times. (**a**) as-quenched, (**b**) pre-oxidated for 30 min, (**c**) pre-oxidated for 60 min, (**d**) vacuum heat-treated for 30 min, (**e**) vacuum heat-treated for 60 min.

The surface morphology was observed after heat treatment, and the results are shown in Figure 5. After heat treatment, the surface roughness of the ribbon became rougher with the extension of time, and the surface roughness affected the corrosion resistance. The rougher the surface, the worse the corrosion resistance [6]. However, due to the amorphous ribbon, the annealing temperature was lower than $T_{x1}$ = 363 °C, the surface of the ribbon itself was defective, the surface roughness had little impact on the corrosion resistance of the ribbon, and the corrosion potential of the vacuum sample moved only slightly to the left. The change in surface roughness of the ribbon after pre-oxidation treatment was more obvious than the change in vacuum, but due to the formation of an oxide layer on the surface of the ribbon, the oxide layer could promote the formation of a passivation film, which could improve the corrosion resistance of the ribbon. The longer the pre-oxidation treatment, the more obviously the oxide layer improved the corrosion resistance of the ribbon.

**Figure 6:**
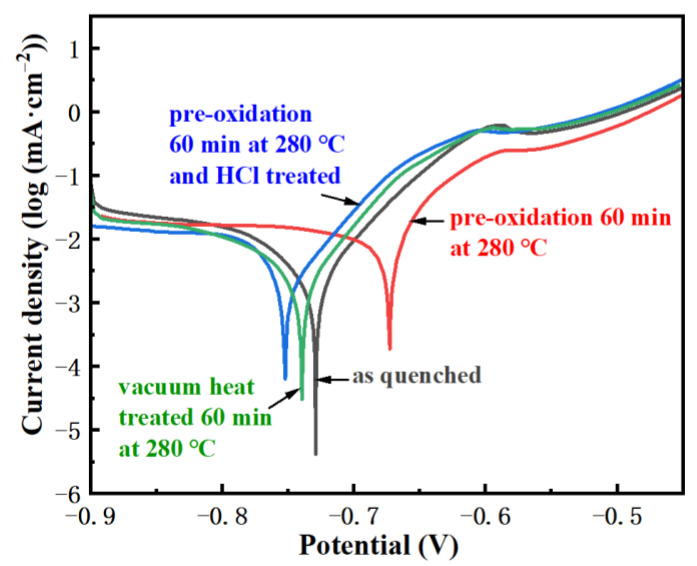
The polarization curve of the sample after removing the surface oxide layer.

In order to further prove that the oxide layer is beneficial to the improvement of the corrosion resistance of the samples, gently wipe the surface of the oxidized sample with a cotton swab dipped in dilute hydrochloric acid. Remove the brown oxide layer on the surface, then rinse off the hydrochloric acid and hydrochloric acid corrosion products with alcohol and ultrapure water, quickly dry the ribbon with cold air, and then measure the sample polarization curve. The result is shown as the curve in Figure 6. It can be seen that, after removing the surface yellow-brown oxide layer, the corrosion potential moves back to the vacuum sample, indicating that the corrosion resistance of the ribbon decreases after removing the oxide layer. The oxide layer has an improving effect on the corrosion resistance; the corrosion resistance of the sample after removing the surface oxide layer decreases worse than the sample after vacuum heat treatment. This might be due to the fact that the hydrochloric acid does not remove all of the oxide layer; only the oxide layer of the surface is removed, and the residual oxide layer fails due to the destruction of the overall structure, and the protective effect fails. On the contrary, it will promote the corrosion reaction.

**Figure 7:**
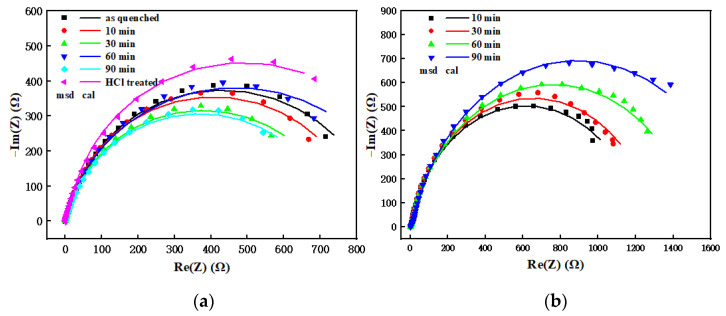
Impedance curve and equivalent circuit diagram of the sample’s heat treatment at 280 °C: (**a**) vacuum, (**b**) non-vacuum.

Figure 7 shows the impedance curve and equivalent circuit diagram of the vacuum and pre-oxidation treatment samples of the amorphous FeCoSiBPC alloy in a 1 mol/L NaCl solution at room temperature. It can be seen that the vacuum and pre-oxidation treatment samples showed capacitive resistance characteristics; with the prolongation of time, the capacitive arc of the pre-oxidation sample was gradually expanded, and the capacitive arc of the vacuum sample was shrunk to varying degrees. The impedance curve was fitted by Zsimwin software (AMETEK, Berwyn, PA, USA), and the equivalent circuit is shown in the figure [7].

Here, $R_{s}$ is the solution resistance between the reference electrode and the working electrode, $QPE_{1}$ and $R_{1}$ represent the capacitance and resistance of the protective layer/corrosion layer, representing the reaction of the interface of the solution and protective layer/corrosion layer [8]; $QPE_{2}$ and $R_{2}$, respectively, represent the double-layer capacitance and charge-transfer resistance due to the deviation caused by the rough and uneven sample surface. The double-layer capacitor *C* is often replaced by the constant phase element *QPE* [9]; the impedance of the *QPE* can be replaced by $Z_{QPE}$:

(1) $$Z_{QPE}=Y_{0}^{-1}\omega^{-n}[\cos\left(\frac{n\pi }{2}\right)-j\sin\left(\frac{n\pi }{2})\right]$$

where $Y_{0}$ is a constant; *ω* is the angular frequency; *n* is the deviation parameter, whose value is 0 to 1, representing the degree to which *QPE* deviates from the capacitance; and *QPE* is equivalent to capacitance *C* when *n* = 1. $QPE_{2}$ and $R_{2}$ represent the capacitance and resistance of electrolyte solutions in electrochemical reactions at the interface of an amorphous alloy [8]. It can be seen from Figure 7 that the $QPE_{1}$ and $QPE_{2}$ of the vacuum-treated sample showed a parallel relationship, and the equivalent circuit diagram of the sample after pre-oxidation treatment was in series, indicating that the passivation film formed by the pre-oxidized ribbon in 1 mol/L NaCl solution was denser than the passivation film formed by the vacuum heat treatment sample, which could play a better protective role [10]. This result was consistent with the polarization curve result.

The fitting results of each component in the equivalent circuit are shown in Table 1. As can be seen from Table 1, the value of the $R_{2}$ was greater than $R_{1}$, which indicates that the inner layer of the passivation film formed was relatively dense, the outer layer was looser, and the inner passivation film played a decisive role in the corrosion resistance of the amorphous alloy [11]. Comparing the *R* values of vacuum and quenched samples, it was found that vacuum heat treatment had less influence on $R_{1}$ and $R_{2}$, and it could be considered that vacuum heat treatment at 280 °C for 10 to 90 min had little impact on the corrosion resistance of ribbon. The *R*-value of the sample after pre-oxidation treatment changed greatly, which meant that the pre-oxidation treatment had a greater impact on the corrosion resistance of the ribbon. The $R_{2}$ increased with the extension of the pre-oxidation time, which suggests that the corrosion resistance of the ribbon had gradually enhanced. This is consistent with the results of the polarization curve [12].

Comparing the vacuum sample and the pre-oxidation sample, the $R_{1}$ of the vacuum sample was greater than the $R_{1}$ of the pre-oxidation sample for 10–60 min, but its $R_{2}$ was less than the $R_{2}$ of the pre-oxidation sample. This indicates that the density of the inner layer of the passivation film increases after the pre-oxidation treatment, and the degree of the densification of the outer layer decreases. The difference between $R_{1}$ and $R_{2}$ after the pre-oxidation treatment was greater than the difference between the vacuum and quenched samples. The reason could be that during the pre-oxidation treatment, oxidation lead to an increase in the roughness of the ribbon surface, resulting in a decrease in the corrosion resistance of the outer layer of the oxide layer. The diffusion of elements would cause corrosion resistance of the inner layer of the oxide layer to increase, and the oxide could promote the formation of a passivation film, which could also increase the corrosion resistance [13]. This was specifically manifested when the value of $R_{1}$ was decreased and when $R_{2}$ rose. Because the corrosion resistance of the inner layer played a decisive role, the overall corrosion resistance of the ribbon was improved. At the same time, comparing the $n_{1}$ values, the $n_{1}$ of the vacuum and as-quenched samples were all around 1, indicating that the thickness uniformity of the protective layer/passivation layer formed was better. The $n_{1}$ values after pre-oxidation treatment had different degrees of deviation, and the uniformity of the thickness of the protective layer/corrosion layer changed, which may be due to the uneven thickness of the oxide layer.

After removing the oxide layer of the pre-oxidized 60min sample with diluted hydrochloric acid (HCl treated in Figure 7a), the equivalent circuit diagram changed from series to parallel mode, and the $R_{2}$ dropped from 1515 ohm to 835.6 ohm. The corrosion resistance of the ribbon after removing the oxide layer dropped to a similar level of vacuum heat treatment, but the surface oxide layer was not completely removed, so its $R_{1}$ value is relatively high.

**Figure 8:**
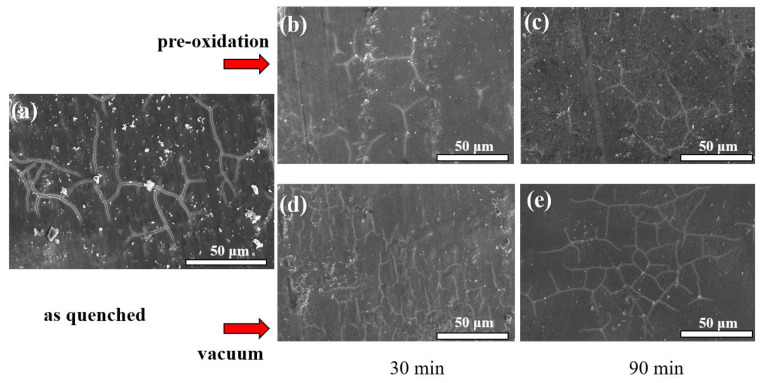
SEM image of the surface after corrosion. (**a**) as-quenched, (**b**) pre-oxidated for 30 min, (**c**) pre-oxidated for 60 min, (**d**) vacuum heat-treated for 30 min, (**e**) vacuum heat-treated for 60 min.

The above Table 1. shows that the oxide layer played a protective role in the ribbon and could promote the formation of the passivation film. We soaked the ribbon in 1 mol/L NaCl solution for 5 days and observed the surface of the corrosive sample. The result is shown in Figure 8 after corrosion; the sample surface appeared granular on the corrosion products, and cracking was observed, which may be caused by the drying of corrosion products. The crack density of vacuum and as-quenched corrosion products was greater than that of the pre-oxidation samples, indicating that the corrosion product layer/passivation layer of the pre-oxidation samples had a high degree of density and strong corrosion resistance.

**Figure 9:**
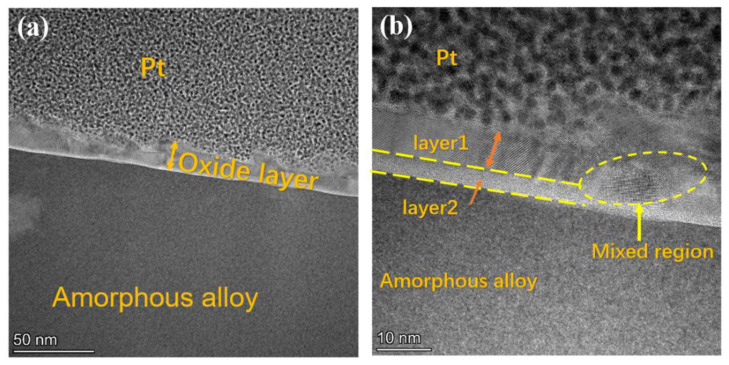
TEM image of the ribbon after pre-oxidation heat treatment at 280 °C for 60 min. (**a**) Surface structure diagram; (**b**) oxide layer structure diagram.

In order to explore the reasons for the improvement of corrosion resistance of ribbon after pre-oxidation treatment, we selected ribbon with the pre-oxidation treatment of 60 min, using FIB to cut the ribbon, and observed the structural morphology of the ribbon oxide layer. In Figure 9a, we can clearly see the presence of the oxide layer, the thickness is about 10–20 nm, the outermost layer is the Pt protective layer sprayed when Fib cutting, and the innermost an amorphous alloy matrix. The thickness of the oxide layer is uneven, and there is a bulge in the outer layer, which is due to defects and unevenness on the surface of the ribbon itself. Figure 9b on the right is an enlarged plot of the oxide layer, which has obvious stratification, which can be expected, and the stratification of the oxide layer has been observed in previous studies [14,15,16,17]. The oxide layer is mainly divided into two layers, layer 1 and layer 2. Layer 2 and the amorphous alloy matrix maintained an amorphous structure; layer 1 was crystallized and nanocrystals were formed (as shown in the mixed region). Nanocrystals have higher corrosion resistance than amorphous alloy [18,19], thereby improving the corrosion resistance of the ribbons.

**Figure 10:**
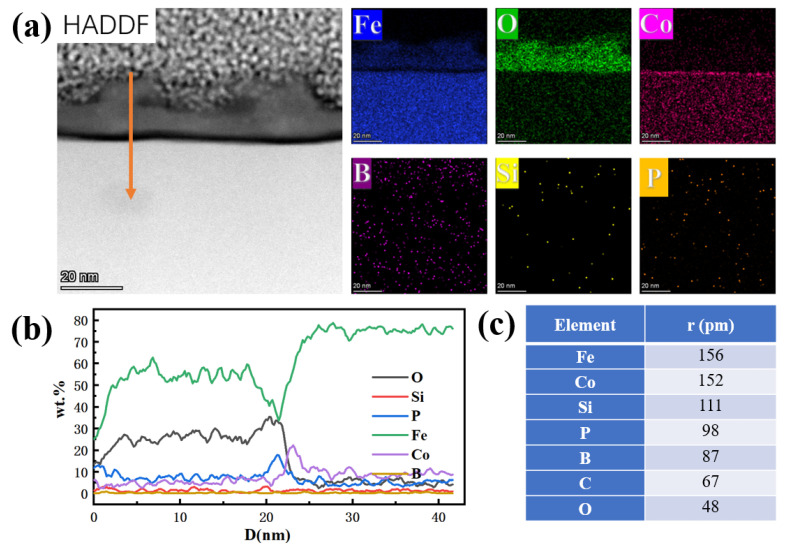
(**a**) TEM image of the surface after pre-oxidation at 280 °C for 60 min; (**b**) results of EDS line scan; (**c**) atomic radius of each element.

Figure 10a is the result of cross-sectional scanning of the oxide layer. The main component of the oxide layer was Fe and its oxides, and there was an aggregation distribution phenomenon at the junction between the oxide layer and the amorphous matrix (oxidation front). The elemental distribution map of Co has a brighter line at the oxidation front. Combined with the EDS line sweep results of (b) (the line sweep direction is shown by the orange arrow in Figure 10a), it can be understood that, in addition to Co, P will also show that there is an aggregation distribution in the oxidation front. The thickness of the aggregation area was about 2–4 nm, which indicated that there was an element diffusion migration phenomenon during the pre-oxidation treatment process. Comparing the content inside and outside the oxidation front of these two elements, the inner Co content was higher than the outer oxide layer, and the element diffusion direction was from the outer layer to the oxidation front diffusion aggregation. On the contrary, the oxide layer content of P was higher than the matrix content; therefore, it was considered that element P was diffused from the internal matrix to the oxidation front. It can be seen from Figure 10c that the atomic radius of Co was large, and diffusion from the outside to the inside could reduce the number of dislocations formed when the outermost oxide layer crystallizes. Due to the aggregation of P and Co, the corrosion resistance of the oxidation front is improved, because the addition of Co and P elements will improve the corrosion resistance of amorphous alloys [20,21,22], but due to its low thickness, after removing the surface oxide layer, the protective effect on the ribbon could not be expressed. Due to the removal of the oxide layer with dilute hydrochloric acid, the overall structure of the oxide layer was destroyed, and the corrosion resistance of the ribbon was reduced.

As there was an element diffusion migration phenomenon, the composition of the oxide layer changed, which could lead to a change of crystallization temperature, and the oxidation caused a drop in crystallization temperature [23,24]. Therefore, crystallization occurred in layer 1 at 280 °C for 60 min.

## 4. Conclusions

(1) After the FeCoSiBPC amorphous ribbon was pre-oxidized at 280 °C, the corrosion resistance of the ribbon in 1 mol/L NaCl solution increased, and the corrosion resistance of the vacuum heat-treated sample at the same temperature was basically unchanged or decreased. Heat treatment changed the roughness of the ribbon surface.
(2) The oxide layer formed by the pre-oxidation treatment had a protective effect on the ribbon and could promote the formation of a dense passivation film. The impedance of the ribbon exhibited dual capacitive impedance characteristics, the as-quenched and the vacuum sample were in parallel, and the pre-oxidation treatment was in parallel.
(3) During the pre-oxidation process, the amorphous ribbon had the phenomenon of element migration, Co migrated from the outside to the oxidation front, and P migrated from the inside to the oxidation front. At the same time, there was a small-scale element (mainly Co and P) aggregation at the junction between the oxide layer and the amorphous matrix, which improved the corrosion resistance of the amorphous alloy.
(4) By studying the effect of pre-oxidation heat treatment on the corrosion resistance of FCoSiBPC amorphous alloy, it had certain guiding significance for the production of amorphous alloy, heat treatment process, and later application.

## Figures and Tables

**Table 1:** Impedance curve fitting data.

Sample		$R_{s}$ (ohm)	$QPE_{1}$ (S·s^n^)	$n_{1}$	$R_{1}\;(\mathrm{ohm})$	$QPE_{2}\;(S\cdot s^{n})$	$n_{2}$	$R_{2}\;(\mathrm{ohm})$
As quenched		0.5254	1.368 × 10^−4^	1	42.6	2.184 × 10^−4^	0.8556	815.9
Vacuum	10 min	0.5120	1.670 × 10^−4^	1	42.42	2.288 × 10^−4^	0.8419	771.9
	30 min	0.4746	1.324 × 10^−4^	1	24.39	2.780 × 10^−4^	0.8218	733.9
	60 min	0.8117	1.124 × 10^−4^	0.9796	10.74	2.468 × 10^−4^	0.8040	932.5
	90 min	0.4274	1.234 × 10^−4^	1	48.55	2.429 × 10^−4^	0.8141	689.4
Pre-oxidation	10 min	1.821	2.81 × 10^−4^	0.8983	1.609	2.617 × 10^−4^	0.8781	1224
	30 min	1.916	4.06 × 10^−4^	0.8533	2.272	2.090 × 10^−4^	0.8785	1299
	60 min	0.6352	1.75 × 10^−4^	0.9070	3.047	1.822 × 10^−4^	0.8467	1515
	90 min	0.7792	7.15 × 10^−4^	0.6059	10.06	1.474 × 10^−4^	0.8514	1756
HCl treated		0.4031	5.587 × 10^−4^	1	127.7	4.210 × 10^−4^	0.9244	835.6

## Data Availability

The data presented in this study are available.
